# Prevalence and characteristics of musculoskeletal complaints in primary care: an analysis from the population level and analysis reporting (POLAR) database

**DOI:** 10.1186/s12875-023-01976-z

**Published:** 2023-02-04

**Authors:** Romi Haas, Alexandra Gorelik, Ljoudmila Busija, Denise O’Connor, Christopher Pearce, Danielle Mazza, Rachelle Buchbinder

**Affiliations:** 1 School of Public Health and Preventive Medicine, Monash University, 553 St Kilda Road, Melbourne, VIC 3004 Australia; 2 Cabrini Health, Malvern, VIC 3144 Australia; 3 Outcome Health, Blackburn, VIC 3130 Australia; 4 Department of General Practice, Monash University, Notting Hill, VIC 3168 Australia

**Keywords:** General practice, Low back pain, Shoulder pain, Knee pain, Neck pain, Big data

## Abstract

**Background:**

Electronic health record datasets have been used to determine the prevalence of musculoskeletal complaints in general practice but not to examine the associated characteristics and healthcare utilisation at the primary care level.

**Aim:**

To describe the prevalence and characteristics of patients presenting to general practitioners with musculoskeletal complaints.

**Design and setting:**

A five-year analysis within three Primary Health Networks (PHNs) in Victoria, Australia.

**Method:**

We included patients with at least one face-to-face consultation 2014 to 2018 inclusive and a low back (≥ 18 years), and/or neck, shoulder or knee (≥ 45 years) complaint determined by SNOMED codes derived from diagnostic text within the medical record. We determined prevalence, socio-demographic characteristics and diagnostic codes for patients with an eligible diagnosis; and number of consultations within one year of diagnosis.

**Results:**

324,793/1,294,021 (25%) presented with at least one musculoskeletal diagnosis, of whom 41% (*n* = 133,279) fulfilled our inclusion criteria. There were slightly more females (*n* = 73,428, 55%), two-thirds (*n* = 88,043) were of working age (18–64 years) and 83,816 (63%) had at least one comorbidity. Over half had a low back diagnosis (*n* = 76,504, 57%) followed by knee (*n* = 33,438, 25%), shoulder (*n* = 26,335, 20%) and neck (*n* = 14,492, 11%). Most codes included ‘pain’ and/or ‘ache’ (low back: 58%, neck: 41%, shoulder: 32%, knee 26%). Median (IQR) all-cause consultations per patient within one year of diagnosis was 7 (4–12).

**Conclusion:**

The burden of MSK complaints at the primary care level is high as evidenced by the prevalence of people with musculoskeletal complaints presenting to a general practitioner, the preponderance of comorbidities and the numerous consultations per year. Identification and evaluation of strategies to reduce this burden are needed.

**Supplementary Information:**

The online version contains supplementary material available at 10.1186/s12875-023-01976-z.

## Background

General practitioners (GPs) are usually the first point of contact with the health care system in developed countries and provide ongoing care for many conditions. In 2017–18, 86% of the Australian population reported visiting their GP multiple times a year [1]. Musculoskeletal problems such as osteoarthritis and back pain are one of the most common reasons for seeking care from a GP [2], with estimates of one in five consultations being for a musculoskeletal complaint [3, 4].

Electronic health records from general practices can be a rich and efficient source of data. Using routinely collected data for research has the potential to improve health outcomes [5]. For example, these data can be used to examine patterns of care to identify where improvements are needed and then to evaluate whether interventions designed to improve care have the desired effect. General practice databases extract and deidentify patient-related information from every GP/patient encounter directly from the electronic medical records of consenting practices. They have been used internationally to examine patterns of care for various conditions including respiratory tract infection, [6] cardiovascular disease [7], chronic hepatitis C [8], chronic kidney disease [9], and diabetes [10–12].

General practice databases have been used to determine the prevalence of some musculoskeletal complaints including arthritis, chronic back pain, gout, osteoporosis, spondyloarthropathies and rheumatoid arthritis in various countries [13–17]. Trends and trajectories of opioid prescription for people with general musculoskeletal conditions, [18, 19] use of osteoporosis medicines in people with osteoporosis [16] and use of biologic drugs in people with psoriatic arthritis and ankylosing spondylitis [20] have also been examined. However, to date the characteristics of patients presenting to general practice with musculoskeletal complaints and the healthcare utilisation at the primary care level has not been comprehensively examined using primary care databases.

This study forms part of a larger project that is using data from the POpulation Level Analysis and Reporting (POLAR) dataset from 2014 to 2018 inclusive to examine patterns of care provided by GPs for people with musculoskeletal complaints [21]. In this paper we describe the prevalence of people with any musculoskeletal complaint and those of the low back, neck, shoulder and knee specifically, and the characteristics of patients who present to GPs with these complaints over a five-year period. We also describe the diagnostic codes and the number of all-cause GP consultations within one year following diagnosis.

## Methods

### Study design and setting

The protocol including a detailed description of the data source, setting, eligibility criteria and diagnostic codes used to identify eligible patients for this study, has been published previously [21]. In brief, this is an analysis of general practice care for patients with musculoskeletal complaints using routinely collected data from the POLAR database. This database contains deidentified patient-related data from electronic medical records of consenting general practices within three PHNs in Victoria, Australia. PHNs are independent organisations funded by the Australian Government to improve patient care by GPs and coordination of care within specific geographic boundaries. At the time of extract, 301 general practices had consented, representing approximately 30% of all general practices within the three participating PHNs. The study population consists of the adult patients attending these practices. Most patients in Australia usually attend the same practice and see the same GP within the practice [22]. In the POLAR database an ‘activity’ occurs anytime a patient record is accessed regardless of whether this was for clinical or administrative purposes. To ensure the ‘activity’ was for a clinical purpose, we restricted the underlying population to adult patients (aged 18 years and over) who had received at least one face-to-face GP consultation between 1/01/2014 and 31/12/2018.

### Participants

We included patients with a low back (≥ 18 years), and/or neck, shoulder or knee complaint (≥ 45 years). Our age criteria were chosen because the prevalence of most musculoskeletal conditions increases markedly after the age of 45 except for low back pain which increases after 18 years [23]. We excluded traumatic diagnoses and other conditions typically primarily managed by a specialist (e.g. inflammatory and autoimmune rheumatic diseases). In Australia, coding is not embedded in the clinical process and needs to be conducted specifically for research purposes. Patients with an eligible musculoskeletal complaint were therefore selected using Outcome Health’s coding of diagnoses according to SNOMED CT-AU terminology, [24] a standardised method for recording medical terms. Clinical natural language processing is used to code narrative text written by GPs within the diagnostic field of the electronic medical record as SNOMED CT-AU terminology. For example, this allowed free-text items such as ‘back pain’, ‘lumbar ache’ and ‘low back pain’ to all sit under the same diagnostic code. Clinical natural language processing conducted by Outcome Health has previously demonstrated accurate coding of over 95% of the narrative text to SNOMED CT-AU terms in a sample of approximately 57,000 diagnosis records [25]. Since every complaint documented within the diagnostic field of the electronic record by a GP will be coded, there can be multiple diagnostic codes entered at the same time. Diagnoses provided by specialists and then documented by a GP will also be coded.

### Variables

The patient-related variables extracted for this study included deidentified patient ID, year of birth, gender, postcode of residence, Statistical Area Level 3 (SA3) of residence [26], date and SNOMED-CT-AU diagnostic codes of eligible musculoskeletal complaint(s), presence of comorbidities and dates of GP consultations on or after the date of first eligible diagnosis until 31/12/2018. A list of eligible diagnostic codes and comorbidities is available from https://clinicalcodes.rss.mhs.man.ac.uk/medcodes/article/174/. Eligible comorbidities included chronic cardiovascular disease, chronic obstructive pulmonary disease, chronic musculoskeletal conditions, cancer, dementia, diabetes, depression/anxiety and obesity that had been present for at least six months prior to the index musculoskeletal complaint [21]. Deidentified practice ID and PHN were also extracted.

### Data analysis

Relevant data were extracted from the POLAR SQL database and imported into Stata V.15 (StataCorp LLC, College Station, TX, USA). Relational data files were systematically merged using patient and practice IDs to select the study cohort. The prevalence of patients with any type of musculoskeletal complaint was calculated as well as those meeting our inclusion criteria. This was the proportion of patients with an eligible age and at least one musculoskeletal diagnostic code out of those with a face-to-face GP consultation during 2014 to 2018 inclusive. The number (%) of patients, general practices and GPs within each PHN was reported.

Socio-demographic characteristics and comorbidities of eligible patients categorised by body region of musculoskeletal complaint included age, gender, residential remoteness according to the Australian Statistical Geography Standard [26], and socio-economic status according to the Index of Relative Socioeconomic Advantage and Disadvantage [27]. Number and type of clinical diagnoses per patient were described according to body region(s) affected and specific SNOMED-CT-AU diagnostic codes. Follow-up period [median (interquartile range (IQR))] and number of all-cause consultations per patient [median (IQR)] within one year of diagnosis were also reported. Median (IQR) were reported as the data were positively skewed. For patients with more than one eligible diagnosis, these data were calculated from the first eligible diagnosis musculoskeletal diagnosis or ‘index’ diagnosis within the study period.

## Results

### Description of study cohort

**Fig. 1:**
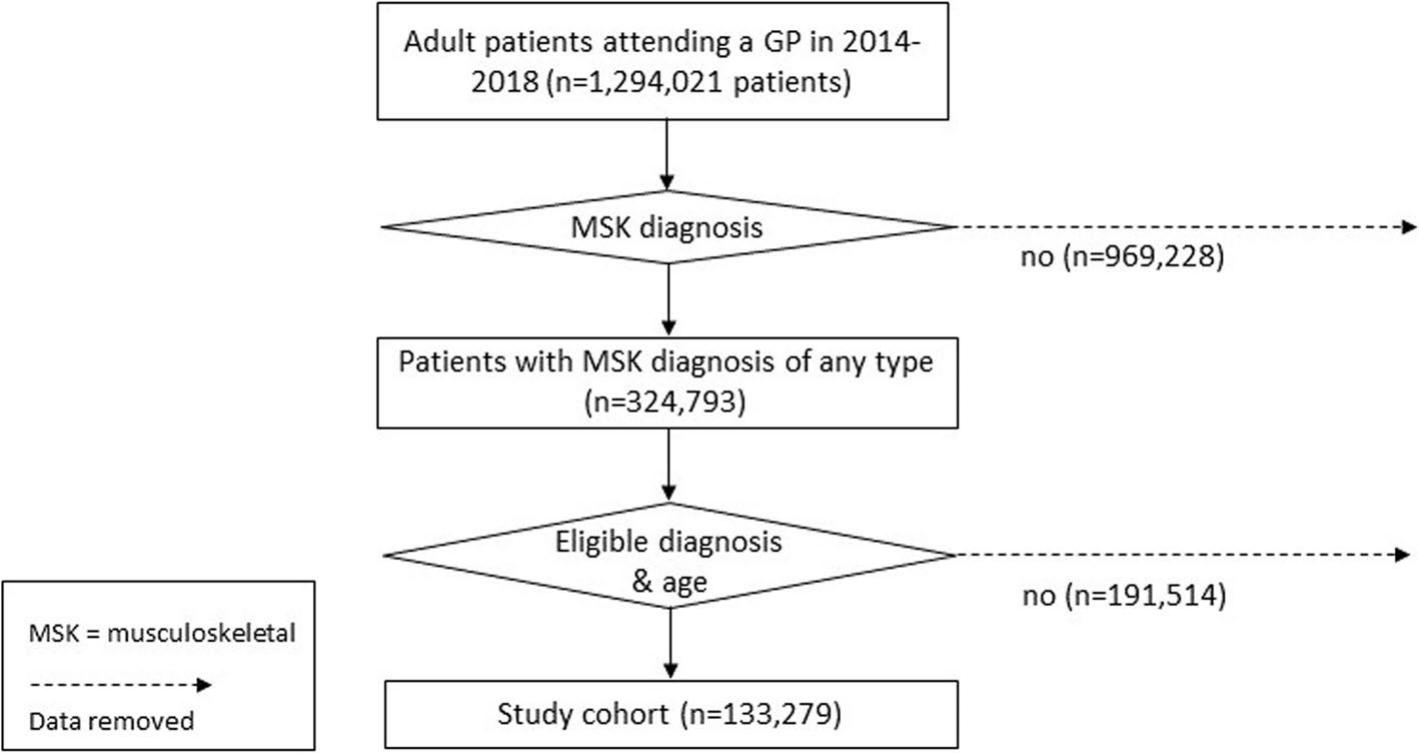
Study cohort flowchart

Selection of the study cohort from the POLAR dataset is presented in Fig. 1. Twenty-five percent (324,793/1,294,021) of adult patients with a face-to-face consultation with a GP during the study period were diagnosed with a musculoskeletal complaint of any type. Of these, 133,279 (41% or 10% of the underlying population) were of eligible age and had a musculoskeletal complaint that fulfilled our inclusion criteria (low back, neck, shoulder and/or knee complaint). 969,228 (75%) patients with a face-to-face consultation and diagnosis did not have a musculoskeletal diagnosis of any type and a further 191,514 (59%) patients with eligible musculoskeletal complaints were outside the age criteria for inclusion.

The included cohort of patients attended 4,538 GPs and 269 general practices (Table 1). The distribution of patients, GPs and general practices within each PHN were approximately proportional. Approximately 50% of patients, GPs and general practices were from Eastern Melbourne PHN, 40% were from South Eastern Melbourne PHN and 10% were from Gippsland PHN. These proportions are also similar to those of the general population living within these areas. Mean (SD) age of the study cohort at index diagnosis was 49.2 (18.5) years for those with low back complaints and 61.9 (12.0), 62.8 (11.8) and 64.2 (11.5) years for those with neck, shoulder and knee complaints respectively. These differences reflect our eligibility criteria where we restricted those with low back complaints to 18 years and over but those with neck, shoulder and knee complaints were restricted to 45 years and over. Fifty-five percent of our cohort were female, 87% lived in a metropolitan location and 11% lived in an area of greatest socioeconomic disadvantage (Table 2). The majority (66%) were of working age (18–64 years) with progressively fewer patients in each 10-year age bracket over 65 years (18.4% 65–74 years, 11.2% 75–84 years and 4.3% 85 years and over). Nearly two thirds (63%) had at least one comorbidity, most commonly cardiovascular (33%), musculoskeletal (27%), and/or depression/anxiety (25%). Median (IQR) follow-up was 2.3 (1–3.7) years.

**Table 1:** Number (proportion) of patients, practices and general practitioners by primary health network

	Study cohort		
	Patients, n (%)	GPs, n (%)	Practices, n (%)
Eastern Melbourne PHN	69,467 (52.1)	2,277 (50.2)	139 (51.7)
South-Eastern Melbourne PHN	53,149 (39.9)	1,561 (34.4)	106 (39.4)
Gippsland PHN	10,663 (8.0)	700 (15.4)	24 (8.9)
**Total**	**133,279 (100)**	**4,538 (100)**	**269 (100)**

*GP* General practitioner, *PHN* Primary health network

**Table 2:** Characteristics of study cohort

	Study population^#^ n	Total study cohort n (%)	Low back^*^ n (%)	Neck^*^ n (%)	Shoulder^*^ n (%)	Knee^*^ n (%)	Multi-site^^^ n (%)
Patients	1,294,021	133,279 (100)	65,612 (49.2)	8,974 (6.7)	18,253 (13.7)	25,264 (19.0)	15,176 (11.4)
Age at diagnosis in years							
18–44	694,705 (53.7)	30,139 (22.6)	29,977 (45.7)	N/A	N/A	N/A	162 (1.07)
45–54	192,397 (14.9)	29,215 (21.9)	11,153 (17.0)	3,025 (33.7)	5,345 (29.3)	5,900 (23.4)	3,792 (25.0)
55–64	170,122 (13.1)	28,689 (21.6)	9,313 (14.2)	2,492 (27.8)	5,328 (29.2)	7,312 (28.9)	4,244 (28.0)
65–74	130,085 (10.1)	24,563 (18.4)	7,554 (11.5)	1,913 (21.3)	4,269 (23.4)	7,014 (27.8)	3,813 (25.1)
75–84	72,814 (5.6)	14,947 (11.2)	5,165 (7.9)	1,127 (12.6)	2,398 (13.1)	3,841 (15.2)	2,416 (15.9)
85 +	33,898 (2.6)	5,726 (4.3)	2,450 (3.7)	417 (4.6)	913 (5.0)	1,197 (4.7)	749 (4.9)
Gender							
Male	554,598 (42.9)	59,692 (44.8)	30,513 (46.5)	3,670 (40.9)	8,170 (44.8)	11,124 (44.0)	6,215 (41.0)
Female	734,789 (56.8)	73,460 (55.1)	35,009 (53.4)	5,297 (59.0)	10,075 (55.2)	14,121 (55.9)	8,958 (59.0)
(Missing)	4,634 (0.4)	127 (0.1)	90 (0.1)	7 (0.1)	8 (0.04)	19 (0.08)	3 (0.02)
Remoteness							
Metropolitan	1,143,398 (88.4)	116,064 (87.1)	57,253 (87.3)	7,883 (87.8)	15,920 (87.2)	21,690 (85.9)	13,318 (87.8)
Inner regional	123,801 (9.6)	14,256 (10.7)	7,048 (10.7)	885 (9.9)	1,906 (10.4)	2,840 (11.2)	1,577 (10.4)
Outer regional	20,024 (1.5)	2,507 (1.9)	1,072 (1.7)	180 (2.0)	373 (2.0)	646 (2.6)	236 (1.6)
Remote	666 (0.05)	53 (0.04)	22 (0.03)	5 (0.06)	5 (0.03)	14 (0.06)	7 (0.05)
Very remote	126 (<0.01)	7 (<0.01)	3 (<0.01)	0 (0.0)	1 (<0.01)	2 (<0.01)	1 (<0.01)
(Missing)	6,006 (0.5)	329 (0.3)	214 (0.3)	21 (0.2)	48 (0.3)	72 (0.3)	37 (0.2)
Socioeconomic disadvantage							
Quintile 1†	122,617 (9.5)	14,685 (11.0)	7,826 (11.9)	900 (10.0)	1,700 (9.3)	2,473 (9.8)	1,786 (11.8)
Quintile 2	156,170 (12.1)	18,196 (13.7)	9,337 (14.2)	1,052 (11.7)	2,365 (13.0)	3,403 (13.4)	2,039 (13.4)
Quintile 3	195,170 (15.1)	23,039 (17.3)	11,156 (17.0)	1,520 (17.0)	3,240 (17.7)	4,326 (17.1)	2,797 (18.4)
Quintile 4	328,456 (25.4)	34,167 (25.6)	16,969 (25.9)	2,198 (24.5)	4,736 (25.9)	6,358 (25.2)	3,906 (25.7)
Quintile 5	484,662 (37.5)	42,786 (32.1)	20,102 (30.7)	3,283 (36.6)	6,161 (33.8)	8,632 (34.2)	4,608 (30.4)
(Missing)	6,195 (0.5)	406 (0.3)	222 (0.3)	21 (0.2)	51 (0.3)	72 (0.3)	40 (0.3)
Comorbidity							
At least 1 comorbidity	480,951 (37.2)	83,816 (62.9)	36,178 (55.1)	5,679 (63.3)	11,916 (65.3)	17,051 (67.5)	12,992 (85.6)
Cancer	29,582 (2.3)	6,759 (5.1)	2,401 (3.7)	525 (5.9)	1,193 (6.5)	1,717 (6.8)	923 (6.1)
Cardiovascular	219,987 (17.0)	43,628 (32.7)	15,150 (23.1)	3,219 (35.9)	7,134 (39.1)	10,656 (42.2)	7,469 (49.2)
Dementia	8,041 (0.6)	1,188 (0.9)	501 (0.8)	79 (0.9)	191 (1.1)	256 (1.0)	161 (1.1)
Depression/anxiety	209,727 (16.2)	33,422 (25.1)	16,527 (25.2)	2,264 (25.2)	4,095 (22.4)	5,164 (20.4)	5,372 (35.4)
Diabetes	75,465 (5.8)	13,939 (10.5)	4,947 (7.5)	926 (10.3)	2,522 (13.8)	3,152 (12.5)	2,392 (15.9)
Musculoskeletal	101,101 (7.8)	36,475 (27.4)	15,438 (23.5)	1,925 (21.5)	4,300 (23.6)	6,373 (25.2)	8,439 (55.6)
Obesity	38,475 (3.0)	8,624 (6.5)	3,756 (5.7)	436 (4.9)	991 (5.4)	1,859 (7.4)	1,582 (10.4)
Respiratory	19,893 (1.5)	4,927 (3.7)	2,050 (3.1)	399 (4.5)	710 (3.9)	859 (3.4)	909 (6.0)
GP consultations during follow-up, n (% within 1 year of diagnosis)	13,255,803 (42.7)	2,295,769 (40.8)	962,558 (43.9)	142,937 (42.6)	294,971 (42.0)	407,309 (40.7)	487,994 (33.5)
All-cause GP consultations per patient in 1^st^ year post diagnosis, median (IQR)	4 (2–8)	7 (4–12)	6 (3–12)	7 (4–12)	7 (4–12)	7 (4–12)	11 (6–17)
Age at diagnosis in years, mean (SD)	45.0 (18.9)	56.5 (17.0)	49.2 (18.5)	61.9 (12.0)	62.8 (11.8)	64.2 (11.5)	63.9 (11.9)
Follow-up period in years, median (IQR)	3.2 (1.6–4.6)	2.3 (1.0–3.7)	2.2 (1.0–3.6)	2.1 (0.9–3.5)	2.1 (1.0–3.5)	2.2 (1.0–3.7)	3.2 (1.9–4.2)

All data presented as n (%) except where stated otherwise

^#^ Study population includes adult patients (aged 18 years and over) who received at least one face-to-face consultation with a GP between 1/01/2014 and 31/12/2018

^*^ Single body region affected by musculoskeletal complaint

^^^ Multiple body regions affected by an eligible musculoskeletal complaint

^†^ A low score indicates relatively greater disadvantage

### GP consultations

The study cohort had a total of 2,295,769 eligible consultations occurring on or after the index diagnosis date during the study period, of which 41% (*n* = 936,512) were within one year of the diagnosis being first recorded. There were a median (IQR) of seven (4–12) all-cause consultations per patient within one year of diagnosis compared to 4 (2–8) consultations per patient in the underlying study population of adult patients consulting a GP within the POLAR database (Table 2).

### Nature of musculoskeletal complaint

**Fig. 2:**
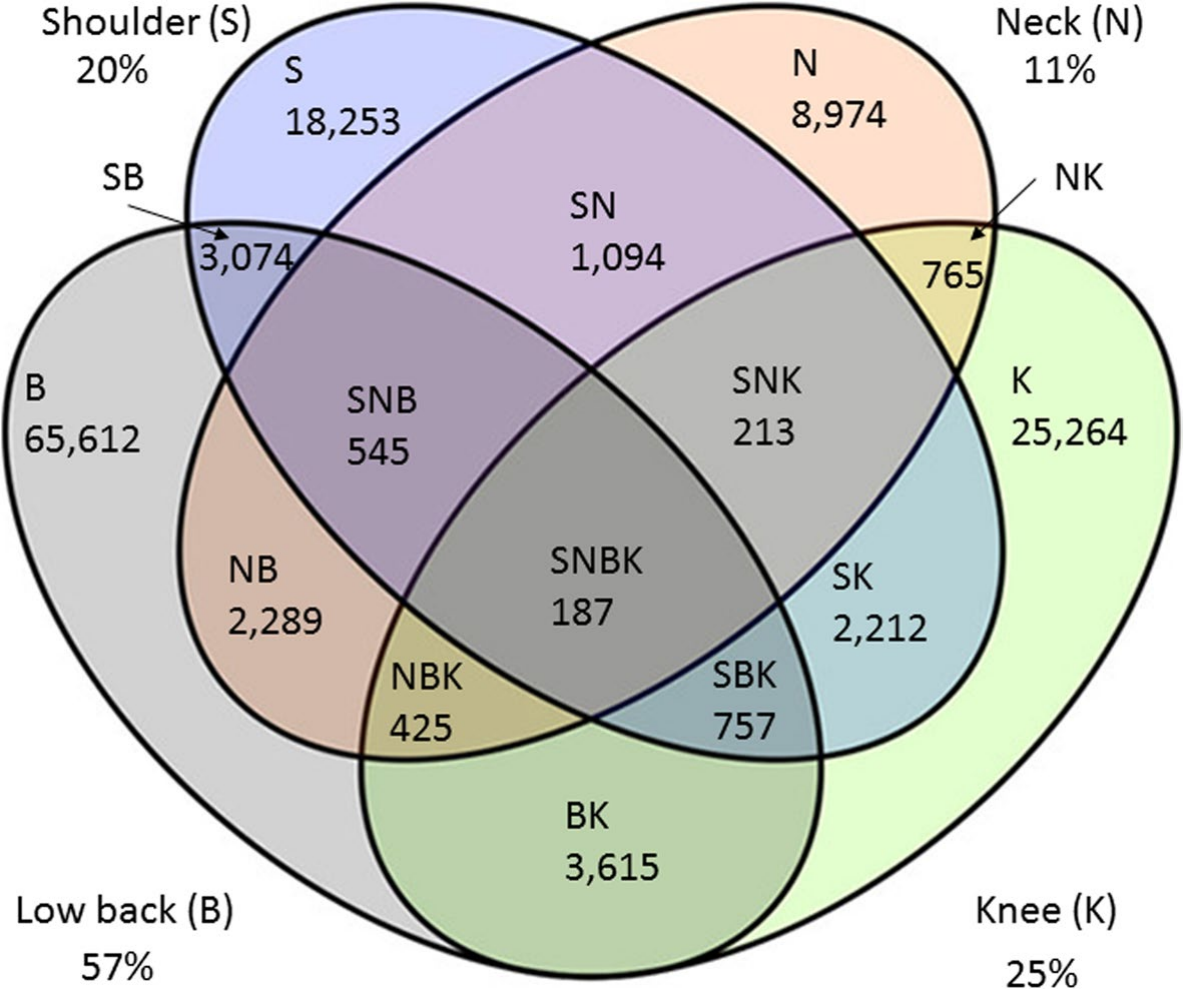
Number of patients by body region affected

Based upon eligible musculoskeletal diagnostic codes, over half of the cohort (*n* = 76,504, 57%) had a low back complaint, a quarter (*n* = 33,438, 25%) had a knee complaint, a fifth (*n* = 26,335, 20%) had a shoulder complaint, and 11% (*n* = 14,492) had a neck complaint (Fig. 2). Almost 90% (*n* = 118,103) had only a single body region complaint, proportionate to the overall body region breakdown (56% low back, 21% knee, 16% shoulder, 8% neck). The remainder had two (*n* = 13,049, 10%), three (*n* = 1,940, 1%) or four (*n* = 187, 0.1%) body region complaints. Of those with multiple body region complaints, the most common combinations were a low back and knee complaint (*n* = 4,984, 33%) and a low back and shoulder complaint (*n* = 4,563, 30%).

Within each body region most patients (*n* = 97,216, 73%) had a complaint labelled with a single diagnostic code [median (IQR) 1 (1–1)], while 22,351 (17%) had two and 13,712 (10%) had three or more diagnoses. The most common diagnostic codes used for each body region are shown in Table 3 and those used in less 1% of diagnostic codes are provided in a supplementary file. There were 12 diagnostic codes used in at least 1% or more of diagnostic codes for low back complaints, 10 codes for neck complaints, 16 codes for shoulder complaints and 13 codes for knee complaints. Backache (46%) and low back pain (12%) were the most common diagnostic codes for low back complaints while neck pain (41%), shoulder pain (32%) and knee pain (26%) were the most common diagnostic codes for the other respective body regions.

**Table 3:** Diagnostic codes (SNOMED CT-AU text) comprising at least 1% of all diagnoses for each body region

	Number (%) of patients labelled with each diagnostic code by body region
**Low back**	
Backache	50,284 (46.2)
Low back pain	12,722 (11.7)
Intervertebral disc disorder	6,946 (6.4)
Degeneration of intervertebral disc	6,154 (5.7)
Chronic back pain	5,559 (5.1)
Spinal stenosis	3,022 (2.8)
Scoliosis deformity of spine	2,368 (2.2)
Fracture of vertebral column	2,237 (2.1)
Osteoarthritis of lumbar spine	2,054 (1.9)
Laminectomy	1,781 (1.6)
Lumbosacral spondylosis	1,439 (1.3)
Injury of back	1,185 (1.1)
**Neck**	
Neck pain	7,792 (41.2)
Cervical spine degeneration	3,806 (20.1)
Cervical radiculopathy	2,421 (12.8)
Cervicogenic headache	925 (4.9)
Cervical disc disorder	755 (4.0)
Torticollis	603 (3.2)
Whiplash injury to neck	507 (2.7)
Cervico-occipital neuralgia	250 (1.3)
Stiff neck	249 (1.3)
Injury of cervical spine	225 (1.2)
**Shoulder**	
Shoulder pain	11,854 (32.1)
Subacromial bursitis	6,236 (16.9)
Rotator cuff syndrome	4,355 (11.8)
Adhesive capsulitis	2,313 (6.3)
Injury of shoulder region	1,873 (4.8)
Supraspinatus tear	1,411 (3.8)
Capsulitis	1,313 (3.6)
Supraspinatus tendinitis	1,053 (2.9)
Impingement syndrome of shoulder region	880 (2.4)
Inflammation of rotator cuff tendon	699 (1.9)
Total shoulder replacement	639 (1.7)
Osteoarthritis of shoulder	596 (1.6)
Arthroscopy of shoulder	569 (1.5)
Subdeltoid bursitis	566 (1.5)
Repair of musculotendinous cuff of shoulder	553 (1.5)
Rupture of tendon of biceps	385 (1.0)
**Knee**	
Knee pain	12,077 (26.4)
Total knee replacement	8,699 (19.0)
Osteoarthritis of knee	8,683 (19.0)
Arthroscopy of knee	2,870 (6.3)
Injury of knee	2,201 (4.8)
Synovial cyst of popliteal space	2,000 (4.4)
Finding of tear meniscus	1,895 (4.1)
Tear of meniscus of knee	782 (1.7)
Acute meniscal tear, medial	719 (1.6)
Prepatellar bursitis	700 (1.5)
Tear of medial meniscus of knee	685 (1.5)
Knee joint effusion	550 (1.2)
Chondromalacia of patella	514 (1.1)

## Discussion

### Summary

Approximately one-quarter of patients attending general practice in three PHNs within Victoria, Australia during the five-year period between 2014 and 2018 had one or more musculoskeletal complaints. Forty-one percent were labelled with at least one low back, knee, shoulder, or neck eligible diagnostic code within our age criteria. There was a slight female preponderance, most people were of working age, had at least one comorbidity and lived in an area of relative socioeconomic advantage. Most commonly the diagnostic code used to label the regional complaint used a non-specific label, i.e., pain or ache. Our study cohort consulted with a GP numerous times within one year of diagnosis and those with multiple body regions affected attended more frequently than those with a single body region affected.

### Comparison with existing literature

The overall prevalence of musculoskeletal complaints among patients consulting a GP in the POLAR database during the study period is consistent with estimates from the 2014–15 Australian National Health Survey which reported that approximately 30% of Australians have at least one musculoskeletal complaint [23] and that the majority of Australians visit their GP multiple times a year [1]. The sex and age breakdown of our cohort is in keeping with Australian Burden of Disease Study 2011 which reported slightly more females in general have a musculoskeletal condition (55%) and 61% of Australians with musculoskeletal conditions are of working age (25–64 years) [28]. Our data are also similar to the Bettering the Evaluation and Care of Health (BEACH) dataset which reported that among patients presenting to general practice with musculoskeletal complaints, 57% are female and 65% are aged 15–64 years [3].

The prevalence of comorbidities in our cohort was in keeping with National Health Survey data that observed 65% of Australians with back pain report having at least one other chronic complaint, of which arthritis (31%), cardiovascular disease (31%) and mental health problems (30%) were most common [23]. The co-occurrence of musculoskeletal complaints with comorbidities, particularly cardiovascular and mental health conditions, is also reported internationally [29, 30].

Our cohort of patients with regional musculoskeletal complaints consulted with a GP for any reason a median of seven times (IQR 4–12) within the first year following diagnosis. This is higher than that of adult patients consulting with a GP within the POLAR database for any reason [median 4 (IQR 2–8)] and that reported by MedicineInsight in the same Australian state during 2017–18 [31]. While this may be partially explained by the older age (due to our eligibility criteria) and female preponderance of our study cohort [31], the high number of GP encounters per year we observed may also be a reflection of the high burden of GP care amongst people with musculoskeletal complaints, especially since our younger cohort of people with back complaints had a similar number of all-cause consultations per year [median 6 (IQR 3–12)] as those with neck, shoulder and knee complaints [median 7 (IQR 4–12)]. This is consistent with an analysis of Australian-wide MedicineInsight data that demonstrated patients with arthritis, chronic back pain, gout, osteoporosis, spondyloarthropathies and/or rheumatoid arthritis attended general practices more frequently than those without these conditions between October 2013 and June 2016 [14]. Our results also suggest patients with multi-site musculoskeletal complaints consult with a GP more frequently than those with a single body region affected (median 11 and 7 occasions/year respectively). While only 11% of our cohort had a diagnosis relating to multiple body regions, this is likely to be an underestimate since we did not include general musculoskeletal terms such as sprain or osteoarthritis that could not be attributed to a specific body region.

Most patients with an eligible musculoskeletal complaint were labelled with a non-specific diagnostic code (e.g., pain, ache) rather than a more specific patho-anatomical or patho-aetiologic label (e.g., cervical radiculopathy, adhesive capsulitis). This is consistent with the non-specific nature of many regional musculoskeletal complaints. For example, in approximately 90% of patients with low back pain, a specific cause of the pain (e.g. disease, structural abnormality or serious injury) cannot be identified [32].

### Implications for research and practice

Relative to the number of musculoskeletal-related SNOMED CT-AU codes available, few codes were used most often. In addition, many of the diagnostic labels describe the same condition (e.g., rotator cuff syndrome, supraspinatus tendinitis, inflammation of rotator cuff tendon), while others describe presumed or actual imaging findings that may or may not be clinically relevant (e.g., degeneration) or a specific treatment rather than a diagnosis (e.g., laminectomy, arthroscopy). This lack of mutually exclusive categories and redundancy has been observed in a study of low back pain coding [33]. Revision of the SNOMED CT-AU classification system to reduce redundancy while ensuring all conditions can be labelled may improve consistency and allow comparison across studies that use this system. GP involvement in assigning diagnostic codes from a list generated in real-time using clinical natural language processing may help to address these issues, but this approach would need to be validated in practice.

There is evidence that the management of patients with musculoskeletal complaints in general practice is suboptimal when compared to recommendations from clinical practice guidelines. Evidence to practice gaps include overuse of imaging, [34, 35] referral to specialist care [36], surgery [37, 38], and opioid prescription [39]. The ongoing use of these low value interventions may contribute to the high burden of GP consultations we observed in this study indicating an urgent need to identify effective strategies to implement evidence-based recommendations for people with musculoskeletal conditions into practice and policy. A 2016 Cochrane review found low quality evidence that guideline dissemination and educational opportunities alone may lead to little or no improvement in guideline-consistent GP care for people with low back pain (7 studies), but that additional strategies such as feedback (2 studies) and GP reminder messages (1 study) may lead to small improvements [40]. The value of audit and feedback is supported by a recent Australian-wide factorial cluster trial that found audit and feedback resulted in > 47,000 fewer selected low back, neck, knee and shoulder imaging tests over 18 months among high requesting GPs [41]. Other promising strategies include digital treatment alogorithms [42], other decision tools [43], and modifying imaging reports to include simple terminology and explicit consideration of context [44].

### Strengths and limitations

The strengths of this study include its large sample size and comparability to population estimates in terms of patient demographics, PHN representation and body regions affected. Limitations of this study include missing data within the POLAR database and the potential for selection bias of consenting practices. Prior to merging the relational data files, nearly 20% of provider files did not include a provider type and 12% of diagnoses records had a missing diagnosis code or missing or implausible diagnosis date. Missing data may occur at random due to a lack of documentation within the electronic health record (responses are not mandated by GPs) or because a code could not be attributed by the clinical natural language process. This means our dataset of patients with musculoskeletal complaints may be incomplete. Despite this, our study cohort appears to be broadly representative of the wider Australian population attending a GP and to those with musculoskeletal complaints within the community. Although approximately 30% of general practices across south-eastern Victoria are included within the POLAR database, non-accredited, corporate-owned practices, and those not using electronic medical records are under-represented. While we do not have the information available to assess for any differences in the characteristics of GPs who were and were not included in this cohort, this potential for difference should be considered when interpreting the frequency of consultations provided by the GPs in this study. Our estimate of the all-cause consultations per year also includes consultations that may be for reasons other than a musculoskeletal complaint. Additionally, our estimate of the prevalence of regional low back, knee, shoulder and neck complaints is likely an underestimate due to our exclusion of selected age groups.

## Conclusion

There is a high burden of patients with non-specific regional musculoskeletal complaints within general practice. Most occur in working age and are accompanied by at least one comorbidity, and many patients attend on multiple occasions. Identification and evaluation of strategies to reduce this burden are needed.

## Supplementary Information

**Additional file 1:** **Appendix 1.** Diagnostic codes used in less than 1% of patients for each body region.

## Data Availability

The dataset supporting the conclusions of this article can be requested from the data custodians, Outcome Health (email:research@outcomehealth.org.au). The statistical analysis code is available from the corresponding author on reasonable request.
