# Supplementary material for: Prevalence and characteristics of musculoskeletal complaints in primary care: an analysis from the population level and analysis reporting (POLAR) database
Source: BMC Prim Care. 2023 Feb 4;24:40. doi: 10.1186/s12875-023-01976-z (PMC9898983; doi:10.1186/s12875-023-01976-z)
Supplement: Supplementary file 1 — Additional file 1: Appendix 1. Diagnostic codes used in less than 1% of patients for each body region. [file 12875_2023_1976_MOESM1_ESM.docx]

**Appendix 1: Diagnostic codes used in less than 1% of patients for each body region**

| **Low back diagnostic codes** | **Neck diagnostic codes** | **Shoulder diagnostic codes** | **Knee diagnostic codes** |
| --- | --- | --- | --- |
| \| Nerve root compression syndrome \| \| --- \| \| Lumbar radiculopathy \| \| Spondylolisthesis \| \| Nerve root disorder \| \| Sacroiliac joint inflamed \| \| Lumbar microdiscectomy \| \| Low back strain \| \| Chronic lower back pain \| \| Spinal arthrodesis \| \| Pain in the coccyx \| \| Chondrectomy of spine \| \| Crush fracture of lumbar vertebra \| \| Sacroiliac joint pain \| \| Lower back injury \| \| Sacral back pain \| \| Lumbosacral spondylosis without  myelopathy \| \| Spondylosis \| \| Lumbar spinal fusion \| \| Disorder of vertebra \| \| Magnetic resonance imaging of  spine \| \| Spasm of back muscles \| \| CT of spine \| \| Arthritis of spine \| \| Fracture of lumbar spine \| \| L4/5 disc \| \| Compression fracture \| \| Spinal stenosis of lumbar region \| \| Prolapsed lumbar intervertebral  disc \| \| Fracture of sacrum \| \| Lumbar discectomy \| \| Sprain of spinal ligament \| \| Compression of lumbar nerve root \| \| Disorder of joint of spine \| \| L5/S1 disc \| \| Spondylolysis \| \| MRI of lumbar spine \| \| Discitis \| \| Lumbar sprain \| \| Radiography of spine \| \| Spinal injury \| \| CT of lumbar spine \| \| Compression fracture of vertebral  column \| \| Degeneration of lumbar  intervertebral disc \| \| Facet joint pain \| \| Lordosis deformity of spine \| \| Lumbar laminectomy \| \| Lumbar spondylolisthesis \| \| Spondylolisthesis L5/S1 level \| \| Sacroiliac arthrodesis \| \| Arthropathy of spinal facet joint \| \| X-ray of lumbosacral spine \| \| Correction of scoliosis \| \| Operative procedure on spinal  structure \| \| Scoliosis of lumbar spine \| \| wedge fracture of vertebra \| \| Diagnostic radiography of coccyx \| \| Spondylitis \| \| Discogenic pain \| \| Curvature of spine \| \| Injury of coccyx \| \| Decompression laminectomy \| \| Back problem \| \| Bone structure of L5 \| \| Fracture of body of vertebra \| \| Lower back structure \| \| Lumbosacral strain \| \| Manipulation of spine \| \| Stiff back \| \| Vertebral osteoporosis \| \| Bone structure of sacrum \| \| Exploration of spine \| \| Lumbar \| \| Pain in lumbar spine \| \| Bone structure of coccyx \| \| CT of lumbar region \| \| Decompression of lumbar spine \| \| Lumbar region back structure \| \| Lumbosacral spine \| \| Spondylosis without myelopathy \| \| Stenosis of intervertebral foramina \| \| Vertebroplasty \| \| Spinal arthritis deformans \| | \| Chronic neck pain \| \| --- \| \| Excision of cervical intervertebral  disc \| \| Neck sprain \| \| Cervical nerve root compression \| \| Kyphosis deformity of spine \| \| Neck injury \| \| Injury of cervical spine \| \| Kyphoscoliosis deformity of spine \| \| Cervical myelopathy \| \| Cervical arthrodesis \| \| Cervical rib \| \| Neck structure \| \| CT of neck \| \| Cervical laminectomy \| \| Strain of neck muscle \| \| MRI of cervical spine \| \| Radiography of cervical spine \| \| CT of cervical spine \| \| Pain in cervical spine \| \| Prolapsed cervical intervertebral  disc \| \| Cervical arthritis \| \| Degeneration of cervical  intervertebral disc \| \| Spinal stenosis in cervical region \| \| Cervical spinal fusion by anterior  technique \| \| Diffuse cervicobrachial syndrome \| \| Cervical radiculitis \| \| Magnetic resonance imaging of neck \| \| Muscle spasm of cervical muscle of  neck \| \| Cervical kyphosis \| | \| Arthritis of acromioclavicular joint \| \| --- \| \| Shoulder reconstruction \| \| Bursitis of shoulder \| \| Arthroscopic shoulder  decompression \| \| Painful arc syndrome \| \| Arthrography of shoulder \| \| Calcific tendinitis of shoulder \| \| Rupture of tendon of biceps,  long head \| \| US shoulder region \| \| Arthroscopic acromioplasty \| \| Injury of glenoid labrum of  shoulder joint \| \| Subluxation of acromioclavicular  joint \| \| Sprain of acromioclavicular ligament \| \| Radiography of shoulder \| \| Osteoarthritis of acromioclavicular  joint \| \| Arthrodesis of shoulder \| \| MRI of shoulder \| \| Entire tendon of supraspinatus  muscle \| \| Full thickness rotator cuff tear \| \| Sprain of shoulder \| \| Repair of shoulder \| \| Shoulder strain \| \| Structure of left shoulder region \| \| Subscapularis tendinitis \| \| Acromioclavicular joint structure \| \| calcific tendinitis \| \| Detachment of the glenoid labrum  and/or capsule of the shoulder joint \| \| Shoulder region structure \| \| Structure of right shoulder region \| \| Structure of rotator cuff including  muscles and tendons \| \| Shoulder tendinitis \| | \| Arthroscopic meniscectomy \| \| --- \| \| Rupture of anterior cruciate  ligament \| \| Tear of lateral meniscus of knee \| \| Derangement of knee \| \| Fracture of tibial plateau \| \| MRI of knee \| \| Disorder of patellofemoral joint \| \| Sprain of knee \| \| Repair of meniscus \| \| Arthroscopy of knee with medial  meniscectomy \| \| Strain of tendon of medial thigh  muscle \| \| Arthritis of knee \| \| Chondrocalcinosis \| \| Patellofemoral osteoarthritis \| \| Total replacement of left knee joint \| \| Suprapatellar bursitis \| \| Rupture of medial collateral  ligament of knee \| \| Total replacement of right knee  joint \| \| Arthroscopic procedure \| \| Patellar tendonitis \| \| Radiologic examination of knee \| \| Haemarthrosis of knee \| \| Arthrodesis of knee \| \| Osteotomy of tibia \| \| Knee locking \| \| Repair of knee cruciate ligaments \| \| Rupture of cruciate ligaments \| \| Sprain of lateral collateral  ligament of knee \| \| Bursitis of knee \| \| Patellectomy \| \| Loose body in knee \| \| Rupture of posterior cruciate  ligament \| \| Swollen knee \| \| Knee region structure \| \| Arthroscopy of knee with lateral  meniscectomy \| \| Replacement of total knee joint \| \| Stabilisation of patellofemoral joint \| \| Aspiration of knee joint \| \| Structure of right knee \| \| Subluxation of patellofemoral joint \| \| Calcium pyrophosphate deposition  disease \| \| Knee joint valgus deformity \| \| Strain of knee \| \| Structure of left knee \| \| Anterior knee pain \| \| Patellar maltracking \| \| Patellofemoral stress syndrome \| \| Repair of anterior cruciate ligament  of knee joint \| \| Arthrotomy of knee \| \| Problem knee \| \| Knee stiff \| \| Arthroscopic lateral patellar release \| \| Osteotomy of proximal tibia \| \| Repair of knee collateral ligaments \| \| Sprain of medial collateral  ligament of knee \| \| Both knees \| \| Contusion of knee \| \| Inflammation of bursa of patella \| \| Knee joint - varus deformity \| \| Repair of patellar tendon \| \| Strain of patellar tendon \| \| Structure of prepatellar bursa \| \| Synovial cyst of knee \| \| Unstable knee \| |

Diagnostic codes presented in order of frequency used
